# Burden, depression and anxiety in primary caregivers of children and adolescents in renal replacement therapy

**DOI:** 10.1590/2175-8239-JBN-2018-0039

**Published:** 2019-02-21

**Authors:** Angélica Godoy Torres Lima, Clécia Cristiane da Silva Sales, Welton Flávio de Lima Serafim

**Affiliations:** 1 Instituto Federal de Educação, Ciência e Tecnologia de Pernambuco Belo JardimPE Brasil Instituto Federal de Educação, Ciência e Tecnologia de Pernambuco, Belo Jardim, PE, Brasil.; 2 Instituto de Medicina Integral Professor Fernando Figueira RecifePE Brasil Instituto de Medicina Integral Professor Fernando Figueira, Recife, PE, Brasil.; 3 Hospital Regional do Agreste CaruaruPE Brasil Hospital Regional do Agreste, Caruaru, PE, Brasil.

**Keywords:** Caregivers, Nephrology, Child, Adolescent, Depression, Anxiety

## Abstract

**Introduction:**

Chronic kidney disease (CKD) is rare in children, but it causes repercussions in several aspects of life, because the disease and treatment cause great changes in the daily lives of the child and his family, increasing the burden on caregivers.

**Objective:**

To evaluate the burden of primary caregivers of children and adolescents who undergo renal replacement therapy (RRT).

**Methods:**

Cross-sectional, observational study performed at the Pediatric Renal Unit of a school hospital in the Northeast. Forty-nine primary caregivers of pediatric patients with CKD in RRT followed up in our clinic participated in the study. We used validated instruments to assess burden, depression and anxiety. We ran some tests to analyze the findings of burden, depression and anxiety in the sample.

**Results:**

Most of the caregivers are the mothers of these children (89.8%), aged between 36 and 45 years (46.9%), have Elementary School education only (55.1%) and reported feeling pain in the body (69.4%), but they did not have chronic disease. The majority of the children have been in RRT from 1 to 3 years (40.8%), aged from 9 to 11 years (30.6%), are male (55.1%), and under hemodialysis (38.8%). The caregivers had a moderate level of burden (2.10), a high prevalence of moderate to severe depression (18.4%) and anxiety (47%), and a strong correlation between burden, depression and anxiety.

**Conclusions:**

Caring for a child with CKD is an intense experience, with negative consequences, due to uncertainties about the future and the very care these children require. We need to do something to help these caregivers better manage care, as well as cope with their own feelings.

## INTRODUCTION

Chronic kidney disease (CKD) in children is different from that in adults, and the largest group of children diagnosed includes congenital anomalies and hereditary diseases. CKD prevalence in children is rare (between 15 and 74.7 cases per million children). The incidence of children and adolescents under dialysis treatment is estimated in 15 patients per million inhabitants. Data collected from registries around the world suggest good survival, even when dialysis is required as early as the neonatal age.^1^^,^^2^^,^^3^

As a chronic condition, CKD can affect children and adolescents, with repercussions in various aspects of life, as the disease and treatment cause great changes in the daily lives of the children and their families, generating difficult moments, with advances and setbacks.^4^

Discussing chronic illness in children is challenging, since we expect the child to grow and develop in the healthiest conditions possible, as opposed to a process involving suffering, pain, and stress. Faced with this aspect, uncertainties and ambiguities arise, as well as the need to balance hope and fear in relation to the new reality that prevails in the daily lives of children, adolescents and their families.^5^

Healthcare professionals need to focus not only on patients' health but also on the health of their caregivers, which are a key element for home care, since patients are more likely not to have their needs met if the caregiver is under high degree of burden.^6^

Such care, because it is very complex, requires preparation in order to provide proper physical and psychological care. The family member usually ends up assuming functions for which he/she is not prepared, and thus ends up having his/her health impaired, also becoming ill.^7^^,^^8^

The burden or stress related to the caregiver role occurs because of the negative emotional and physical responses of a caregiver to the changes and demands in the process of helping someone with a physical or mental disability. It is a multidimensional concept that encompasses the psychological, health, social, and economic aspects of care delivery. burden is characterized by the amount of time and assistance devoted to dealing with the consequences of a disability.^8^^-^10

Caregiving has important implications for the long-term well-being of the patient, since caregivers play a key role in preserving the benefits of rehabilitation, which is optimized if the family is healthy and caring.^9^^,^^10^

This study aimed to evaluate the burden and estimate the prevalence of depression and anxiety in primary caregivers of children and adolescents who undergo renal replacement therapy in a school hospital in the metropolitan region of Recife, in addition to identifying possible factors associated with the outcome variables.

## METHODS

We carried out a cross-sectional, descriptive, observational and quantitative study, which evaluated 49 main caregivers of patients enrolled in the Renal Replacement Therapy (TRS) program of the Pediatric Renal Unit of the Instituto de Medicina Integral Professor Fernando Figueira (IMIP), corresponding to the population of children and adolescents from 1 to 15 years of age, in any of the RRT modalities offered by the service (hemodialysis, peritoneal dialysis and renal transplantation) between October and December 2016.

One of the inclusion criteria in the study was to be the primary caregiver of the child or adolescent enrolled in the RRT program. Caregivers of children/adolescents in dialysis therapy were excluded due to exacerbation of chronic renal disease in conservative treatment and those who did not know about the health-disease process of the child or adolescent.

We consulted the patients' charts and structured interviews with selected primary caregivers. We did not use a sample calculation, in order to perform a census-type study, since the total population corresponded to only 58 caregivers; however, there were 9 losses: 3 eligible individuals refused to participate and 6 others did not attend the scheduled appointments during the data collection period.

We invited the caregivers to participate in the study, and explained its purpose and the confidentiality of the information given. They participated in the interview after signing the informed consent form. The study was approved by the local Ethics Committee under the CAAE number of 58487716.0.0000.5201.

We used a structured questionnaire to collect information, based on references in the literature about the topic addressed, created by the researchers; besides the use of instruments validated for Brazilian Portuguese. We used the Caregiver Burden Scale (CBS) to assess caregivers burden, which was broken down into 5 domains: general tension, isolation, disappointment, emotional involvement and environment. The burden score in each domain is obtained by calculating the average score of associated items, being classified in three levels: low (1-1.99), moderate (2-2.99) and severe (3-4).

We used the Beck Depression Inventory (BDI) and the Beck Anxiety Inventory (BAI), which are reliable instruments with good psychometric properties, for depression and anxiety screening. Both consist of self-assessment scales composed of 21 items, including symptoms and behaviors, with intensities ranging from 0 to 3 - the higher the value, the greater the severity of the symptoms - in which the total score comprises a range from 0 to 63. Beck et al. recommend the following cutoff points for the BDI: less than 10 = no depression or minimal depression; 10 to 18 = mild to moderate depression; from 19 to 29 = moderate to severe depression; from 30 to 63 = severe depression. For BAI, the level of anxiety should be classified as follows: 0 to 7 = minimum anxiety; 8 to 15 = mild anxiety; 16 to 25 = moderate anxiety; and 26-63 = severe anxiety.^11^^,^^12^^,^^13^^,^^14^

We built a Microsoft Excel spreadsheet for data analysis, which we exported to the SPSS software, version 18. We calculated and built the respective frequency distributions to evaluate the socio-demographic profile and the presence of comorbidities, and we used the chi-square to compare the proportions found in the levels of the evaluated factors.

We assessed the burden using the mean of the score for each domain. For depression and anxiety, we calculated the means of the score and the prevalence for the classifications of the evaluated levels. We used the Kolmogorov-Smirnov test to assess the normality of the burden, depression and anxiety scores. When normality was indicated we employed the Student's t-test and ANOVA. In cases where normality was not indicated, we employed the Mann-Whitney test and the Kruskal-Wallis test.

After the bivariate analysis, we checked for correlation between the burden score and the depression and anxiety scores. For this evaluation we used the Spearman correlation test. We then adjusted a linear model to determine an expression to estimate the burden score from the depression and anxiety scores. The level of significance was 5%.

## RESULTS

Concerning the distribution of the socio-demographic data of the caregivers evaluated, as depicted on Table 1, the majority were female (93.9%) and the mother of the patient (89.8%). Most of these caregivers were married (69.4%), aged between 36 and 45 years (46.9%) and had only Elementary School education (55.1%). More than half of the study population (67.3%) stated that they did not perform any type of paid work (57.6% of caregivers left employment to care for the patient); the average monthly family income was R $ 1,804.00. About one-third (36.7%) had cared for the child/adolescent with CKD for more than 6 years. The test was significant in all factors evaluated, except for the "time taking care of the child/adolescent with CKD (*p*-value = 0.201) factor", indicating that the number of caregivers is similar among the categories.

**Table 1:** Distribution of the socio-demographic data of the caregivers of children under Renal Replacement Therapy

Factor employed	N	%	*p*-value
Gender			
Male	3	6.1	< 0.001
Female	46	93.9	
Age			
24 to 35 years	19	38.8	0.014
36 to 45 years	23	46.9	
46 to 56 years	7	14.3	
Schooling			
Illiterate (0)	2	4.1	< 0.001
Basic (1 to 9)	27	55.1	
Medium (10 to 12)	16	32.7	
Higher (more than 12)	4	8.2	
Marital status			
Single	12	24.5	< 0.001
Married	34	69.4	
Divorced	3	6.1	
Caregiver-patient relation			
Mother	44	89.8	< 0.001
Father	1	2.0	
Grandmother/grandfather	2	4.1	
Other	2	4.1	
Work			
Yes	16	32.7	0.015
No	33	67.3	
Why does not work			
Left the job to care	19	57.6	0.001
Housewife	12	36.4	
Retired	2	6.1	
How long has been taking care of the child/adolescent with CKD			
Up to 1 year	8	16.3	0.201^1^
More than 1 to 3 years	13	26.5	
More than 3 to 6 years	10	20.4	
More than 6 years	18	36.7	
Monthly income			
Minimum - maximum	300 - 10000	-	
Mean ± standard deviation	1804 ± 1733	-	
Child's age			
Less than 9 years	10	20.4	0.752
9 to 11 years	15	30.6	
12 to 13 years	13	26.5	
14 to 15 years	11	22.4	
Child's gender			
Male	27	55.1	0.475
Female	22	44.9	
Child's schooling			
Illiterate	4	8.2	< 0.001
1 to 5 years	33	67.3	
6 to 10 years	12	24.5	
Current treatment			
Hemodialysis	19	38.8	0.679
Peritoneal dialysis	14	28.6	
Transplant	16	32.7	
Time in RRT			
Up to 1 year	14	28.6	0.531
More than 1 to 3 years	20	40.8	
More than 3 years	15	30.6	

Most of the children in RRT had been in it for more than 1 and up to 3 years (40.8%); they were between 9 and 11 years old (30.6%); males (55.1%); had 1 to 5 years of schooling (67.3%) and were currently undergoing hemodialysis therapy (38.8%). This larger number of patients on hemodialysis occurred due to losses (1was under peritoneal dialysis and 8 were transplanted) during the data collection period. The proportions comparison test was significant only in the child's schooling factor (*p*-value < 0.001), indicating that the schooling of 1 to 5 years was the most prevalent factor in the study sample.

When questioned about the presence of chronic pain, the majority of caregivers stated that they experienced pain in their bodies (69.4%), with the spine being most affected (45.5%), legs (20.5%) and arms (18.2%), the remaining 15.9% referred to several other parts of the body. In addition, 73.5% of them believed that their bodies had undergone changes resulting from the care of the child/adolescent with CKD, and 83.7% stated that they had changes in their emotional states after starting to care for the patient (data not shown).

Hypertension (16.3%), dyslipidemia (6.1%), diabetes mellitus (2%) and depression (2%) were the most prevalent diseases. The proportion-comparison test was significant in all factors evaluated (*p*-value < 0.001 for all), indicating that the number of caregivers without comorbidities was significantly higher than those who had some type of comorbidity (data not shown).

Table 2 shows the distribution of the Caregiver Burden Scale score for the assessment of the caregiver burden according to the following domains: general tension, isolation, disappointment, emotional involvement, environment and total burden. It is noteworthy that, in descending order, the caregiver presents greater burden in factors such as general tension (mean = 2.33), isolation (mean = 2.15) and disappointment (mean = 2.12). The domains with lower levels of burden were: emotional involvement (mean = 1.49) and environment (mean = 1.97).

**Table 2:** Distribution of the Caregiver Burden Scale mean score to assess caregiver burden

Factor assessed	Mean	Standard deviation	*p*-value
Overall tension	2.33	0.92	
Isolation	2.15	0.95	
Disappointment	2.12	0.83	< 0.001
Emotional involvement	1.49	0.78	
Environment	1.97	0.66	
Total burden	2.10	0.66	-

^1^p-value in the Friedman test (if the *p*-value < 0.05 the burden level differs among the domains).

Table 3 shows the distribution of the Beck's depression and anxiety scales. We notice that most caregivers did not have depression nor had a minimum level of depression (63.2%). We also noticed that the proportional comparison test was significant (*p*-value < 0.001), indicating that the number of caregivers without depression is significantly higher than the others.

**Table 3:** Frequency of depression and anxiety in caregivers according to BDI and BAI

SCALE	N	%	*p*-value^1^
BDI			
No depression	31	63.2	< 0.001
Mild symptoms	9	18.4	
Moderate symptoms	4	8.2	
Severe symptoms	5	10.2	
BAI			
Minimum anxiety	13	26.5	0.908
Mild anxiety	13	26.5	
Moderate anxiety	13	26.5	
Severe anxiety	10	20.5	

1 *p*-value of the proportions comparison test (if the *p*-value < 0.05 the percentage values of the levels pertaining to the assessed factors are different).

As for anxiety (Table 3), we found the same proportion of patients with minimal, mild and moderate anxiety (both categories with 26.5%). For severe anxiety, the percentage of caregivers was 20.5%. The proportion-comparison test was not significant (*p*-value = 0.908), indicating that the distribution of the degree of anxiety among the caregivers is homogeneous.

We used the mean and standard deviations of the outcome variables: burden, depression and anxiety to evaluate possible association with the characteristics of caregivers, patients and the treatment of CKD by means of hypothesis tests. In addition to the variables presented in Table 1, the following were compared: profession, having some religion, receiving some financial help, if the caregiver has any illness, if the caregiver has some type of chronic pain, time spent in daily childcare , if the child has had conservative treatment before and another type of TRS has been performed.

Among the variables evaluated in the distribution comparison test, only the chronic pain variable was significant when compared to depression (*p*-value = 0.024), indicating that the only factor that presents association and significantly increases the level of caregiver's depression is chronic pain. This same variable and the others mentioned above were not significant when compared to burden and anxiety (all presented a *p*-value greater than 0.05) (data not shown).

Table 4 shows the correlation between the burden, depression and anxiety scores to assess the influence of depression and anxiety as factors associated with increased caregiver burden. We noticed that the burden has high and significant correlation with depression and anxiety. Furthermore, we noticed that depression and anxiety are also highly correlated. When adjusting the linear regression model for the caregiver's burden, an increase of one unit of the depression score is inferred to increase 0.044 points in the burden score (constant = 1.628 and *p*-value < 0.001). The anxiety score did not remain in the model because of its high correlation with the depression score.

**Table 4:** Assessing the correlation among the burden, depression and anxiety scores

Correlations	r	*p*-value
Burden x Depression	0.745	< 0.001
Burden x Anxiety	0.560	< 0.001
Depression x Anxiety	0.750	< 0.001

Pearson's correlation test. r = correlation level.

## DISCUSSION

The profile of caregivers is congruent with that of participants in other studies with female predominance in the role of primary caregiver, mainly performed by the child's mother.^15^^,^^16^^,^^17^^,^^18^ This predominance is explained by the sociocultural role attributed to the female figure as the leading provider of family care. Most of the time, the activity is carried out with full dedication, with the mother, in addition to the other required care, being the only member of the family responsible for going to the hospital and staying with the child.^16^^,^^19^ As primary caregivers, mothers accept greater responsibility than the other members of the family and have more attributions of care than the parents.^18^^,^^20^

The average age of the parents is within the age group of 36 to 45 years, most have low schooling, Elementary School only, they are low-income families and the mother does not work. The source of income is guaranteed by the father, who earns around a minimum wage, and income is complemented by social programs.^16^^,^^18^^,^^20^

Cimete^16^ reported that the majority of the children/adolescents were male, had had the disease for an average of 5.6 years and had been treated by hemodialysis on an average of 3.1 years, resembling our results. The age of children/adolescents varied significantly, from children under 1 year all the way to 17 years, in several studies, due to the rarity of the disease in children/adolescents and because there are no major differences in the manifestation of the disease according to the age group.^17^ In our study, parents of adolescents above 15 years of age were not interviewed, since their kids are transferred to other services shortly after reaching this age.

Our findings showed that the average caregiver's burden index was 2.10, considered a moderate level. The maximum and minimum values of burden were 2.33 and 1.49 in the domains of general stress and emotional involvement, respectively. The general tension assesses physical and emotional disorders induced by the responsibility of the care, problems related to the care and the time necessary to perform care activities. Using this same scale, Piran et al.^18^ and Medynska et al.^20^ found an average overhead of 1.92 and 2.39, respectively. The authors reported the highest care burden in general tension, averaging 2.26 and 2.32, respectively.

Among the factors reported in the literature that increase the burden of caregivers of children/adolescents with CKD, there are: the number of drugs used by the child/adolescent, especially the injectables; frequent hospital visits; need to carry out complex procedures often; children/adolescents more dependent on daily activities; reduction of social activities and smaller support networks, such as help from other people, whether family or friends.^15^^,^^21^^,^^22^

Our study did not correlate the level of education of caregivers with burden. However, other studies indicate that mothers with a higher education level reported a lower burden of care, since higher education promotes parental confidence in the management of their children's health problems.^23^ Education is an important source of capacity, since it affects the perception of caregivers regarding stressful factors, promotes their ability to solve problems and mental flexibility, positively affecting their quality of life.^18^

Studies have reported that caregivers suffering from a chronic disease do not present a greater burden than the healthy ones; however, the fact of feeling some pain in the body is associated with depressive symptoms in the caregivers who participated in our study. The results of another study showed that chronic caregivers have more stress, and they have a higher disappointment score.^24^

The type of RRT did not influence burden increase or reduction, including Tong et al.,^22^ who reported that transplantation did not necessarily reduce care burden, rather the nature of concerns and difficulties were different.

Parents of children with CKD report lower quality of life, difficulties in managing child care, higher levels of anxiety and maladaptive behavior.^17^^,^^21^ burden among caregivers can have very negative consequences, among them a strong correlation was found between burden with depression and anxiety (Table 4), corroborating another study that demonstrated that caregiver burden was a strong predictor for depression and anxiety in parents of children with health problems.^25^

There is a high prevalence of moderate to severe depression symptoms (18.4%) and anxiety (47%) when compared to the general population. Data from the WHO (2017)^26^ estimate that approximately 4.4% of the world population suffers from depression, and 3.6% from anxiety disorders. Considering the Brazilian population, depression and anxiety-related disorders affect 5.8% and 9.3% of Brazilians, respectively. Other studies report that the rates of depression and anxiety are higher in caregivers of chronic patients than in the general population.^19^^,^^27^

The literature demonstrates that among the factors that may contribute to the depression and anxiety of the parents of children/adolescents with CKD are: limitations in their social lives imposed by the disease on parents and children, such as little time for leisure and other restrictions; 16^-^17^,^^21^ parents' concern about the appearance, development, and future prospects of their children; physical, emotional and social changes that the disease causes in the child; financial difficulties, transportation and accommodation during prolonged hospitalization.^16^^,^^21^ Most parents think that they will lose their children, which creates a lot of stress because they live with this permanent emotional uncertainty about the child's future and the CKD evolution.^16^^-^17

Although some studies cite the financial difficulties due to low income and the short time for leisure activities due to the time of dedication of the caregiver to the child/adolescent with CKD as factors that may predispose to depression and anxiety in caregivers, our study did not find associations between burden, depression and anxiety with the income and time spent as a caregiver variables. In addition, we did not find associations between increased burden, depression and anxiety and the other socio-demographic variables of our study; these are also not mentioned as risk factors in other studies with children/adolescents with CKD.^15^^-^18^,^^20^^-^22

In addition to the psychological consequences, overburdened caregivers also have problems with poor health habits, and end up falling ill in the long term,^15^ because they tend to neglect their own health needs and forego preventive medical visits, as well as not having a healthy diet nor practicing physical activities. The child's needs and care are more important, even to the detriment of the parents' own health.^15^^,^^17^

In pediatric care, efforts are commonly focused on providing treatments that address needs and monitor the well-being of the affected child, rather than considering and including parents as intervention targets to indirectly benefit the child and make explicit that parents require attention for themselves, when considering the caregiver-patient binomial.^21^

Javalkar et al.^15^ suggest that interventions and programs to improve patient care management can help alleviate the caregiver's burden, which may be an important secondary outcome to be assessed by such programs. These strategies to improve intrapersonal well-being should aim to reduce parental anxiety and increase their confidence to manage the care of a sick child.

Current methods to reduce caregiver burden include interventions to increase disease awareness, psycho-educational programs, and educational interventions in self-care, stress management, and communication skills.^15^^,^^22^ Another strategy is the creation of support groups to reduce the feeling of isolation, in addition to socialization and learning coping skills from other parents in relation to nutrition and health care management.^16^^,^^22^

## CONCLUSION

Caring for a child with CKD is an intense experience that has negative consequences because of uncertainties about the future and the care that these children require. This is expressed through the average level of burden identified in these caregivers, along with the high prevalence of depression and anxiety in this population. Through the hypothesis tested, we found no statistical significance correlating factors associated with the increase of burden, anxiety and depression in these caregivers, except for the "body pain" variable, which influenced the depression outcome. There were no major changes in the levels of burden, depression and anxiety among caregivers of children/adolescents submitted to different RRT modalities, since they varied only the nature of their concerns and difficulties.

As positive aspects of this study, we mention the concomitant evaluation of burden, depression and anxiety through validated scales in the same population; in addition to the fact that we did not find quantitative approach studies in the Brazilian literature involving caregivers of pediatric patients submitted to the three RRT modalities, which has not been reported in the Brazilian reality.

The limitations of this study consist of the small population studied and the fact that it was carried out in a single center, thus compromising its generalization to other regions or socioeconomic contexts, making it necessary to carry out other multicentric studies with larger populations, so that more comprehensive conclusions can be drawn. Another limitation is the fact that the prevalence of depression and anxiety found did not apply to a diagnosis, since tracing instruments were used.

The prevalence of depression and anxiety in this population of caregivers is worrying, and it is necessary to implement programs and actions that aim to help them better manage child care, as well as their own feelings.
